# Unusual Cause of Respiratory Distress in a Term Neonate

**DOI:** 10.31486/toj.21.0101

**Published:** 2022

**Authors:** Aymen Mirza, Maribel Martinez, Sasikumar Kilaikode

**Affiliations:** Department of Pediatrics, Ochsner Louisiana State University Health-Shreveport, Shreveport, LA

**Keywords:** *Infant–newborn*, *respiratory distress syndrome–newborn*, *surfactant dysfunction*

## Abstract

**Background:** Respiratory distress is a clinical finding often seen in neonates. Common causes of respiratory distress in this population include respiratory distress syndrome, transient tachypnea of the newborn, infection, aspiration, and cardiac etiologies. We present the case of a neonate who presented with respiratory distress with no identifiable cause on initial workup. The patient was eventually found to have a variant of a genetic mutation that predisposed the infant to this presentation.

**Case Report:** A term male infant born via spontaneous vaginal delivery was admitted to the pediatric service at 3 weeks of age because of tachypnea. Chest x-ray showed perihilar infiltrates. Septic screen, thyroid function test, sweat test, echocardiogram, intracranial ultrasound, and modified barium swallow were normal. Computed tomography scan of the chest showed ground glass opacities in the upper and lower lobes. Airway evaluation showed no evidence of obstruction or anatomic abnormalities. Bronchoscopy showed no masses or tracheomalacia. Bronchoalveolar lavage was negative for infection. The infant was treated with intravenous antibiotics, steroids, and furosemide but continued to be tachypneic and required supplemental oxygen. Genetic studies were obtained to assess for surfactant deficiencies, and the patient was transferred to another center for a higher level of care. Genetic evaluation was positive for NKX2.1 variance mutation C.190C. The patient's symptoms improved, and he was weaned to room air by 3 months of age.

**Conclusion:** When evaluating a child with unexplained pulmonary disease, clinicians should have a high index of suspicion for interstitial lung disease including surfactant protein mutations.

## INTRODUCTION

Respiratory distress is common in neonates but typically improves with treatment during an expected period. Common causes of respiratory distress in term neonates are respiratory distress syndrome, transient tachypnea of the newborn, infection, persistent chemical pneumonia from meconium aspiration, structural respiratory anomalies, and cardiac etiologies.^1^ Children who present with respiratory distress may have other symptoms including nasal flaring, grunting, tachycardia, tachypnea, cough, and other nonspecific symptoms^2^; thus, a thorough evaluation must be conducted to determine the underlying cause. We present the case of a 3-week-old term infant who presented with respiratory distress requiring supplemental oxygen with no identifiable cause on initial workup. The patient was eventually found to have a genetic mutation causing surfactant dysfunction, predisposing the infant to this presentation.

## CASE REPORT

**Figure 1.**
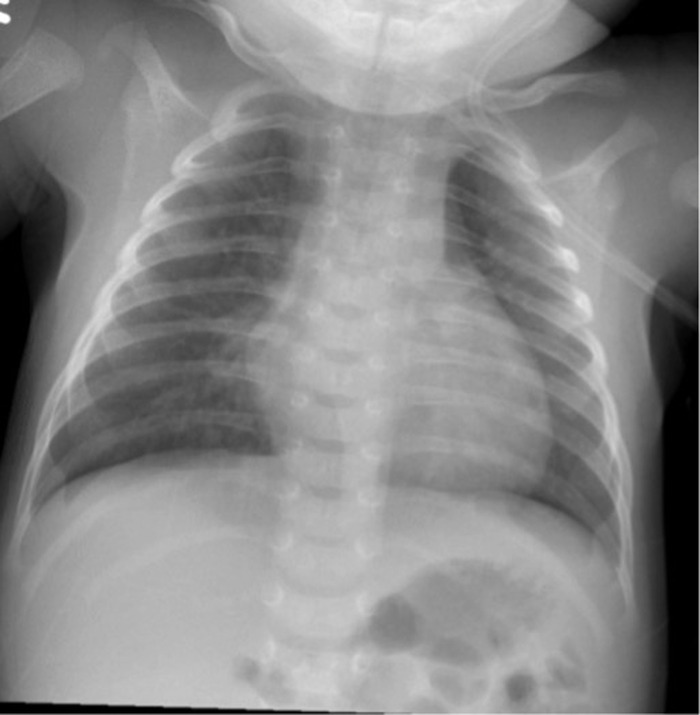
Chest radiograph showed bilateral perihilar hazy infiltrates that were greater on the right.

A term male infant born via spontaneous vaginal delivery was admitted to the pediatric service at 3 weeks of age because of tachypnea. Perinatal history was unremarkable except for meconium-stained fluid; the infant was discharged home on day of life 2. At the patient's 3-week well visit, his mother reported a 2-day history of increased work of breathing and dry cough. Review of systems was negative for stridor, fever, vomiting, sweating with feeds, or apneic episodes. Initial physical examination was significant for a respiratory rate of 88/min, with increased work of breathing. Radiography of the chest showed bilateral perihilar infiltrates that were greater on the right than the left (Figure 1). Brain natriuretic peptide (BNP) was elevated at 895 pg/mL (reference range, 0-99 pg/mL). Echocardiogram showed a patent foramen ovale, ejection fraction of 70% to 75%, and no evidence of pulmonary hypertension. Intracranial ultrasound obtained to evaluate other causes of elevated BNP was normal. Infectious workup including blood count, C-reactive protein (CRP), and blood culture was negative. The patient was discharged home after 4 days without apparent cause for his tachypnea.

**Figure 2.**
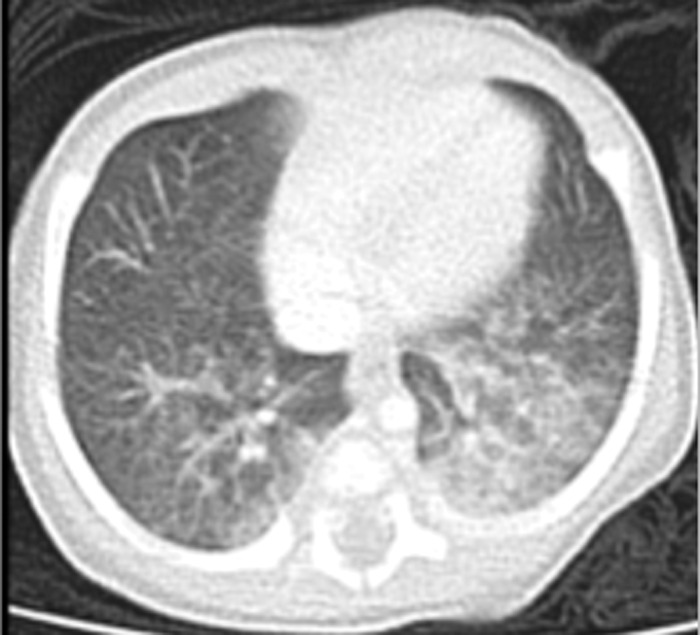
Computed tomography of the chest showed patchy bilateral pulmonary ground glass abnormalities in the upper and lower lobes.

The patient was readmitted 1 week later because of persistent tachypnea (respiratory rate of ≥90/min), new onset hypoxemia (89% to 93% on room air), and retractions. Cardiology, pulmonology, and otolaryngology specialists were consulted. Septic screen, metabolic panel, and thyroid function tests were negative. Sweat chloride test was negative. Computed tomography (CT) scan of the chest showed ground glass opacities in the lower lobes (Figure 2). Modified barium swallow study ruled out aspiration, and contrast esophagram was normal. Airway evaluation showed no evidence of obstruction or anatomic abnormalities. Bronchoscopy showed no masses, lesions, or tracheomalacia. Bronchoalveolar lavage was negative for infection.

The infant was treated empirically with intravenous piperacillin/tazobactam 80/10 mg/kg 3 times daily for 5 days, oral azithromycin 10 mg/kg daily for 1 day followed by 5 mg/kg daily for 4 days, 1 dose of oral furosemide 2 mg/kg, and prednisolone 1 mg/kg twice daily for 5 days. He required supplemental oxygen via nasal cannula during the entire 15-day hospital stay. Most of the common underlying etiologies for respiratory distress were ruled out. In view of the patient's clinical presentation and CT scan, interstitial lung disease was suspected. Genetic studies for surfactant protein mutations were obtained before the patient was transferred. The genetic evaluation was positive for NKX2.1 variance mutation C.190C.

The patient was transferred to a higher center for further management and eventually discharged home on oxygen. No further testing was done at the higher center as the infant's clinical condition was improving. He was followed closely by a pediatric pulmonologist and pediatrician. Aside from his tachypnea and hypoxemia, his growth was appropriate. No further symptoms developed. Oxygen therapy via nasal cannula was continued until about 3 months of age when he was successfully weaned off the oxygen, and his symptoms resolved. The patient's parents report that the infant is well, has not needed oxygen therapy, and has no signs of respiratory distress or hospitalizations.

## DISCUSSION

Genetic mutations of NKX2.1, previously known as TTFI (thyroid transcription factor 1), on chromosome 14q13 have an autosomal dominant pattern of inheritance and have been associated with brain-lung-thyroid defects.^3^ Our patient did not have any pertinent family history which might be explained by the fact that this syndrome is characterized by a highly variable penetrance and expressivity; it can involve only 1 organ system or any combination of all 3, including neurologic manifestations, pulmonary disease, and congenital hypothyroidism.^4^ Neurologic manifestations include hypotonia that can progress to chorea and/or ataxia. Pulmonary involvement, the second most common manifestation, can include respiratory distress syndrome in neonates, interstitial lung disease in young children, and pulmonary fibrosis in older patients.^5^ Hypothyroidism associated with this gene mutation can be attributed to decreased thyroid regulator production and development.^5^

In our patient, no neurologic or thyroid abnormalities were identified, and the family denied any family history; the child presented solely with respiratory symptoms despite a negative workup that ultimately could be explained by NKX2.1 gene variability. Identifying the mutation in our patient led to relief for the family in knowing the diagnosis and will enable screening and possible early identification of endocrine or neurologic manifestations.

In the pulmonary system, homeobox NKX2.1 protein expression can cause a rare form of progressive respiratory failure that is highly correlated to altered surfactant production.^6^ NKX2.1 protein expression regulates the development of lung structures by regulating respiratory epithelial cell genes and, thus, is important in surfactant protein metabolism. Genetic variants of NKX2.1 have been associated with decreased surfactant production contributing to neonatal respiratory distress syndrome and the development of interstitial lung disease.^6^ The decreased surfactant causes increased alveolar surface tension predisposing to atelectasis, increased ventilation-perfusion mismatch, and increased pulmonary inflammatory response that increase the potential for lung injury. In severe cases, disrupted lung development is a likely mechanism for the respiratory disease.^7^ Histopathology findings support the hypothesis that disruption of NKX2.1 targets functional and structural lung development and surfactant homeostasis.^7-10^ The finding of ground glass opacities on chest CT scan in our patient and his continued respiratory distress may be explained by NKX2.1 gene dysfunction and correlated to decreased surfactant production resulting from altered protein metabolism.^6^

In children with NKX2.1 gene mutation, repeated episodes of respiratory distress and decreased lung immunity can lead to increased vulnerability to lung infections.^4^ In most patients with this gene mutation, mechanical ventilation is required to maintain adequate lung function at birth.^3,4,7-9^ Further research indicated that hydroxychloroquine, in addition to azithromycin and prednisolone, is another treatment modality that can be used to improve lung function in patients who present with respiratory distress and lung infections.^11^ Hydroxychloroquine has been associated with improved surfactant protein production; however, further research is required because this association is currently indeterminate.

Our patient did not require mechanical ventilation, but he was treated with azithromycin, oral steroids, and supplemental oxygen. Although evidence is limited that this treatment combination was the reason for the improvement in our patient's symptoms, we can speculate that it played a part in improving the underlying lung disease.

## CONCLUSION

An in-depth evaluation is required in term infants who present with recurring respiratory symptoms when all other causes of tachypnea and hypoxia have been ruled out. Despite its rarity, clinicians should have a high index of suspicion for pulmonary disease caused by surfactant protein mutations when evaluating a child with unexplained respiratory symptoms. NKX2.1 is a genetic defect that can potentially cause interstitial lung disease and should be suspected in neonates with hypothyroidism or neurologic abnormalities; however, respiratory distress secondary to this mutation can present even without hypothyroidism or neurologic abnormalities.

## Notes

*This article meets the Accreditation Council for Graduate Medical Education and the American Board of Medical Specialties Maintenance of Certification competencies for Patient Care and Medical Knowledge.*
